# Increased Presence of Cerebral Microbleeds Correlates With Ventricular Enlargement and Increased White Matter Hyperintensities in Alzheimer’s Disease

**DOI:** 10.3389/fnagi.2020.00013

**Published:** 2020-01-31

**Authors:** Takeshi Kuroda, Motoyasu Honma, Yukiko Mori, Akinori Futamura, Azusa Sugimoto, Satoshi Yano, Ryuta Kinno, Hidetomo Murakami, Kenjiro Ono

**Affiliations:** ^1^Division of Neurology, Department of Medicine, Showa University School of Medicine, Tokyo, Japan; ^2^Department of Physiology, Showa University School of Medicine, Tokyo, Japan; ^3^Department of Internal Medicine, Showa University Northern Yokohama Hospital, Kanagawa, Japan; ^4^Department of Neurology, The Jikei University School of Medicine, Tokyo, Japan

**Keywords:** Alzheimer’s disease, cerebral microbleeds, glymphatic system, paravascular drainage, perivascular drainage

## Abstract

**Objective**: To investigate whether the number of cerebral microbleeds (CMB) could be a useful indicator to predict glymphatic system dysfunction in Alzheimer’s disease (AD) patients, by comparing the degree of cerebral spinal fluid (CSF) and interstitial fluid (ISF) stasis.

**Methods**: Forty probable AD patients were included, with those exhibiting two or more CMB were included in the multiple CMB group (mCMB, *n* = 21, mean = 11.1), and none or one CMB included in the non-multiple CMB group (nmCMB, *n* = 19, mean = 0.84). CMB was defined in axial gradient recalled echo (GRE) T2*-weighted images. Evans index (EI) was calculated to measure lateral ventricle enlargement, Voxel-based Specific Regional Analysis System for Alzheimer’s Disease (VSRAD) software was used to determine the extent of gray and white matter atrophy, and Fazekas scale (FS) was used to determine white matter hyperintensities (WMH).

**Results**: EI was significantly larger in mCMB than in nmCMB, while the gray and white matter volume was not different between groups. Thus, the difference in lateral ventricle enlargement between AD with and without multiple CMB reflects a combination of the degree of brain atrophy and the extent of CSF stasis. FS was higher in mCMB than in the nmCMB, suggesting the failure of ISF elimination was more severe in mCMB cases.

**Conclusion**: The difference in lateral ventricle enlargement and WMH between AD with or without multiple CMB may reflect a difference in the degree of CSF/ISF stagnation.

## Introduction

The lymphatic drainage system is essential for the maintenance of water and solute balance, homeostasis, metabolism, and immunity within the tissue. This system is made up of a network of blind-ended capillaries that drain into larger vessels. These vessels are responsible for removing the lymph from the interstitial fluid (ISF) that surrounds tissues and most organs, which contains waste materials, fluid, proteins, and cells (Dissing-Olesen et al., 2015). The central nervous system has its own unique lymphatic drainage structure, comprised of a basement membrane-based perivascular pathway, a brain-wide glymphatic pathway, and cerebrospinal fluid (CSF) drainage routes, including sinus-associated meningeal lymphatic vessels and olfactory/cervical lymphatic routes (Sun et al., 2018). Drainage of extracellular fluids, particularly CSF/ISF, is not only important for volume regulation, but also for the removal of waste products such as amyloid-β protein (Aβ; Bakker et al., 2016). The glymphatic system, a recently discovered glial-dependent CSF/ISF system for waste clearance, is composed of a network of peri- and para-vascular spaces throughout the brain (Sun et al., 2018). It is often assumed that Aβ is cleared from the cerebral ISF into the CSF, with a specific role for peri- and para-vascular spaces as drainage pathways from the brain parenchyma (Bakker et al., 2016). The movement of CSF/ISF has important implications for understanding basic physiological processes (Simon and Iliff, 2016).

An impairment of the glymphatic system has been identified in Alzheimer’s disease (AD; Roher et al., 2003). The peri- and paravascular pathways are believed to play crucial roles in the glymphatic system, with vessel pulsations and patent blood flow appearing to be driving forces for lymphatic drainage along artery walls (Carare et al., 2008; Iliff et al., 2013). Atherosclerosis and stiffness of small vessels lead to decreased vascular pulsation, which then reduces peri- and paravascular glymphatic circulation (Weller et al., 2009; Iliff et al., 2013). Moreover, cerebral hypoperfusion in a mouse model of AD leads to accelerated accumulation of Aβ in the walls of leptomeningeal vessels (Okamoto et al., 2012). The surrounding amyloid deposits are also thought to destroy the vascular walls and block perivascular spaces, exacerbating the impairment of glymphatic drainage (Roher et al., 2003).

Increased white matter hyperintensities (WMH) in AD, observed in both magnetic resonance imaging (MRI) fluid-attenuated inversion recovery (FLAIR) and T2-weighted imaging, are also important findings that suggest an impairment of the glymphatic system. Although previous studies proposed that WMH reflects axonal loss and demyelination following chronic ischemia due to small vessel disease (SVD), recent studies have suggested that WMH is also a consequence of failure to eliminate ISF from white matter (Carare et al., 2013; Benjamin et al., 2014; Prins and Scheltens, 2015). Furthermore, the presence of WMH is common in idiopathic normal pressure hydrocephalus (iNPH), which is characterized by gait disturbance, cognitive impairment, and urinary incontinence, along with radiological evidence of ventriculomegaly out of proportion to cortical atrophy, and is attributed to an impairment of CSF circulation (Bradley, 2001; Johansson et al., 2016). WMH in iNPH can often be reversed by shunting treatment (Tullberg et al., 2001). These results suggest that stagnation of CSF circulation can also disturb ISF circulation, and consequently, improving CSF circulation may improve ISF circulation.

The most common age-related cerebral SVD subtypes are: (1) arteriolosclerosis and related processes affecting deep perforating arteries; and (2) cerebral amyloid angiopathy (CAA), which is the result of Aβ deposition in the walls of small to medium vessels in the cortex and leptomeninges (Pantoni, 2010; Charidimou et al., 2012). CAA and hypertensive microangiopathy (HM) are major pathological mechanisms for cerebral microbleeds (CMB) in AD (Jellinger, 2002; Hanyu et al., 2003; Cordonnier et al., 2006; Yamada, 2015). CMB are small perivascular hemosiderin deposits resulting from leakage through small cerebral vessels, visualized by gradient-recalled echo (GRE) T2*-weighted MRI. These neuroimaging features are observed in individuals with various etiologies of dementia, including AD, cerebrovascular disease, and normal aging (Fazekas et al., 1999; Greenberg et al., 2009). Lobar microbleeds reflect CAA, whereas non-lobar microbleeds are associated with HM (Poels et al., 2010; Benedictus et al., 2013). CAA-related microbleeds are an important histopathological feature that reflects vascular disorder in AD and is likely caused by vessel fragility and rupture due to Aβ deposition (Yamaguchi et al., 1992). Non-lobar microbleeds may reflect HM, based on vascular risk factors such as age, smoking, and elevated blood pressure (Benedictus et al., 2013). Although CAA and HM are often both present in AD, the frequency of CMB is an indicator of vascular impairment in AD pathogenesis, regardless of their localization.

We hypothesized that CMB due to CAA and HM reflect the condition of the vasculature, and indicates the presence of an impairment of peri- and paravascular lymphatic drainage in AD. In this study, we investigated whether the number of CMB detected could be a useful indicator to predict the degree of underlying glymphatic system dysfunction in AD, comparing the degree of CSF/ISF stasis in AD with or without multiple CMB.

## Methods

### Patients

We included 40 probable AD patients (mean age: 78.90; SD: 7.94; range: 56–92; 22 females) out of 72 patients, who between January and December 2018 received a consultation for memory loss at the memory clinic of the Division of Neurology in the Department of Medicine at the Showa University School of Medicine. Diagnosis of probable AD was based on the diagnostic guidelines of the National Institute on Aging-Alzheimer’s Association (NIA-AA) workgroups for AD (McKhann et al., 2011). Patients with other dementias and normal cognition were excluded. All patients underwent dementia screening, including medical history, neurological and physical examination, cognitive assessment, blood examination, and brain MRI. This study was approved by the Ethics Committee of Showa University School of Medicine. All subjects gave written informed consent in accordance with the Declaration of Helsinki.

### MRI Data Acquisition

Structural MRI scans were conducted using a 1.5T MR scanner (Magnetom Essenza, Siemens, Germany). All patients underwent a scan protocol including T1-weighted, fluid-attenuated inversion recovery (FLAIR) and GRE T2*-weighted imaging. The slice thickness of each image was 6 mm.

CMB was defined as small round foci of hypointense signal in the brain parenchyma, measuring up to 5 mm on GRE axial T2*-weighted images (Wardlaw et al., 2013). CMB was counted throughout the brain and divided into: (1) lobar CMB, within the cerebral cortex/subcortex (frontal, parietal, temporal, occipital); and (2) non-lobar CMB, within the deep white matter (DWM), thalamus, basal ganglia, brainstem, and cerebellar cortex.

Evans index (EI: maximum width of the frontal horns of the lateral ventricles/maximal internal diameter of cranium at the same level) was calculated on FLAIR axial images. The width was measured on three consecutive slices in each section, and then the slice with the largest diameter at the maximal width of the frontal horns was selected. In the same slice, the largest internal diameter of the cranium was measured (Evans, 1942; Brix et al., 2017).

Analysis of MRI images was performed using Voxel-based Specific Regional Analysis System for Alzheimer’s Disease (VSRAD^®^ advance 2, Eisai, Japan) software to determine the extent of gray and white matter atrophy (Matsuda, 2016). VSRAD software is widely used in the clinical diagnosis of AD in Japan. VSRAD is software that quantitatively calculates the extent of brain atrophy (percent of volume reduction in gray and white matter) compared to an MRI imaging database of 80 age-matched healthy controls, based on voxel-based morphometry (VBM). *Z*-scores [(normal control average of voxel-level − patient’s voxel-level)/(normal control standard deviation)] is calculated in each voxel, and the areas with a *Z*-score ≥ 2 are considered atrophied. The extent of atrophy is expressed as percent volume loss. Using this software, we calculated the percentage of hippocampal gray matter atrophy (% HGM), whole-brain gray matter atrophy (% BGM), and whole-brain white matter atrophy (% BWM) in all patients.

To assess WMH on FLAIR weighted axial image, we used the Fazekas scale (FS), which is used to quantify the amount of white matter hyperintense lesions (Fazekas et al., 1987, 1993). This scale divides the white matter into periventricular white matter (PVWM) and DWM, and each region each is given a grade from 0 to 3 depending on the size and confluence of lesions as follows: PVWM: 0 = absent, 1 = caps or pencil-thin lining, 2 = smooth halo, 3 = irregular periventricular signal extending into the deep white matter; DWM: 0 = absent, 1 = punctate foci, 2 = beginning confluence, 3 = large confluent areas. We used a total score ranging from 0 to 6 by summing the PVWM and DWM scores to evaluate WMH.

### Neuropsychological Examination

Cognitive profile was assessed in all patients using the Clinical Dementia Rating (CDR; Morris, 1993), Mini-Mental State Examination (MMSE), and Hasegawa’s Dementia Scale-Revised (HDS-R; Folstein et al., 1975; Imai and Hasegawa, 1994). HDS-R is a scale initially set for the purpose of screening dementia, which has been widely accepted both for clinical use and epidemiological surveys in Japan.

### Risk Factors for SVD

Laboratory testing was performed to evaluate lipid metabolism (TC: total cholesterol, LDL-C/HDL-C: high-density and low-density lipoprotein ratio, TG: triglycerides), glucose metabolism (HbA_1C_: hemoglobin A1c), the presence of heart failure (BNP: brain natriuretic peptide), and renal function (eGFR: epidermal growth factor receptor). The presence of hypertension was determined based on self-reported medical history and/or medication use. Apolipoprotein E (ApoE) genotype, a risk factor AD and CAA were identified by Invader assay.

### Statistics

Unpaired *t*-tests were performed to detect differences between means, and chi-square tests were performed to analyze ratios. All tests were two-tailed. The results are presented as the mean ± standard deviation (SD). The level of statistical significance was defined as *p* < 0.05. SPSS 22.0 for Windows (IBM, Inc., Chicago, IL, USA) was used for the statistical analyses.

## Results

**Figure 1:**
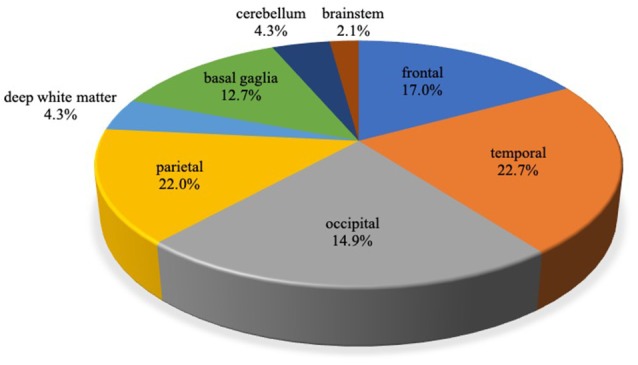
Localization of cerebral microbleeds (CMB) in Alzheimer’s disease (AD). A total of 141 CMB were detected. Of these, 108 (76.6%) were lobar and 33 (23.4%) were non-lobar. CMB localized in the cerebral cortex or subcortex of frontal, temporal, occipital, the parietal lobe was defined as lobar CMB, and CMB localized in deep white matter (DWM), basal ganglia, cerebellum, and brainstem was defined as non-lobar CMB.

Among 40 probable AD patients, 141 CMB were detected in total. One-hundred and eight (76.6%) were lobar, and 33 were non-lobar. The location of lobar CMB was 22.3% in the frontal lobe, 29.6% in the temporal lobe, 28.7% in the parietal lobe, and 19.4% in occipital lobe (Figure 1). Patients with two or more CMB were grouped under the multiple CMB group (mCMB, *n* = 21, mean = 11.1), and patients with none or one CMB were grouped under the non-multiple CMB group (nmCMB, *n* = 19, mean = 0.84). Twenty patients had restricted lobar CMB. Patients with zero or one restricted lobar CMB were grouped under the non-multiple lobar CMB group (nmLCMB, *n* = 18, mean = 0.44), and patients with two or more restricted lobar CMB (matched probable CAA upon modified Boston criteria, Linn et al., 2010) were grouped under the multiple lobar CMB group (mLCMB, *n* = 12, mean = 7.42; Table 1). No patients had cortical superficial siderosis or primary intracerebral hemorrhage.

**Table 1:** Comparison of parameters between Alzheimer’s disease (AD) with non-multiple and multiple cerebral microbleeds (CMB).

	nmCMB (*n* ≤ 1)	mCMB (*n* ≥ 2)	*p*-value	nmLCMB (*n* ≤ 1)	mLCMB (*n* ≥ 2)	*p*-value
Number	19	21		18	12
Mean age	76.58	81.00		76.50	78.83	
Ratio of women (%)	68.42	42.86		72.22	33.33	
**Neuropsychological examination**
CDR	1.42	1.67	0.21	1.44	1.08	0.14
MMSE	19.84	21.38	0.34	19.89	22.92	0.06
HDS-R	18.11	18.55	0.81	18.44	21.00	0.12
**Genetic evaluation**
ApoE ε4 (%)	52.63	47.62	1.00	50.00	58.33	0.94
**Brain MRI investigation**
Evans index	0.28	0.31	0.004**	0.28	0.32	0.006**
Fazekas scale	2.79	4.05	0.016*	2.83	3.58	0.24
VSRAD analysis						
%HGM extent	54.17	50.87	0.69	51.90	53.97	0.83
%BGM extent	7.80	6.40	0.10	7.61	6.50	0.28
%BWM extent	4.95	5.18	0.79	7.35	8.84	0.85
**SVD risk factors**
TC (mg/dl)	224.26	194.52	0.033*	227.61	197.58	0.06
LDL-C/HDL-C	2.08	2.10	0.92	2.11	2.02	0.74
TG (mg/dl)	150.68	122.76	0.24	152.61	126.58	0.39
HbA1c (%)	6.24	6.44	0.52	6.26	6.53	0.49
eGFR (ml/min/1.73m2)	62.41	65.68	0.56	61.39	69.95	0.21
BNP (pg/ml)	41.17	89.16	0.07	40.93	80.28	0.13
Hypertension (%)	52.63	86.71	0.039*	50.00	83.33	0.07

Evans index (EI) was significantly larger in mCMB and mLCMB compared to nmCMB and nmLCMB, and the Fazekas scale was significantly higher in mCMB compared to nmCMB. On the other hand, there was no significant difference in %HGM, %BGM and %BWM extents calculated by VSRAD analysis between groups. In brief, AD with multiple CMB had enlarged lateral ventricle and severe WMH compared to AD without multiple CMB, but there was no significant difference in brain gray and white matter volume between groups. nmCMB, non-multiple cerebral microbleeds; mCMB, multiple cerebral microbleeds; nmLCMB, non-multiple lobar cerebral microbleeds; mLCMB, multiple lobar cerebral microbleeds; CDR, clinical dementia rating; MMSE, mini-mental state examination; HDS-R, Hasegawa dementia rating scale revised; ApoE, apolipoprotein E; %HGM, hippocampus gray matter atrophy; %BGM, brain gray matter atrophy; %BWM, brain white matter atrophy. *p < 0.05, **p < 0.01.

**Figure 2:**
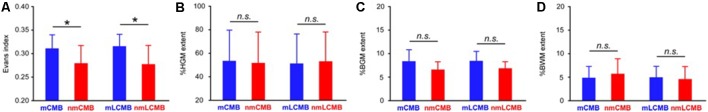
Comparisons of Evans index (EI) and brain atrophy in Alzheimer’s disease (AD) with and without multiple CMB. **(A)** EI in the multiple CMB group (mCMB) group was significantly larger than that in the non-multiple CMB group (nmCMB) group, and similarly, the multiple lobar CMB group (mLCMB) group was larger than the non-multiple lobar CMB group (nmLCMB) group. There was no significant difference between the mCMB and nmCMB groups or between the mLCMB and nmLCMB groups in **(B)** percentage of hippocampal gray matter atrophy (% HGM), **(C)** whole-brain gray matter atrophy (% BGM), or **(D)** whole-brain white matter atrophy (% BWM). The results suggest that AD with multiple CMB exhibit significantly enlarged lateral ventricles compared to AD without multiple CMB, whereas no significant difference in brain atrophy was found between groups. Asterisks indicate significant differences (*p* < 0.05). Error bars indicate SD. n.s., not significant.

The EI in the mCMB group was significantly larger than that in the nmCMB group (*t*_(38)_ = 2.977, *p* = 0.004), and the EI in the mLCMB was significantly larger than that in the nmLCMB group (*t*_(28)_ = 1.210, *p* = 0.006; Figure 2A). No patients showed disproportionately enlarged subarachnoid-space hydrocephalus (DESH) suggesting iNPH (Hashimoto et al., 2010). VSRAD analysis showed that there was no significant difference in the mean % HGM (*t*_(38)_ = 0.399, *p* = 0.692), % BGM (*t*_(38)_ = 1.671, *p* = 0.103), or % BWM (*t*_(38)_ = 0.264, *p* = 0.793) between the nmCMB and mCMB groups, or between the nmLCMB and mLCMB groups (% HGM: *t*_(28)_ = 0.221, *p* = 0.827; % BGM: *t*_(28)_ = 1.103, *p* = 0.279; % BWM: *t*_(28)_ = 0.188, *p* = 0.852). These results suggest that there was no difference in the percentage of gray and white matter atrophy (indicating *Z*-score ≥ 2) between groups (Figures 2B–D). Unpaired *t*-tests revealed that FS in the mCMB group was significantly higher than that in the nmCMB group (*t*_(38)_ = 2.525, *p* = 0.016), while there was no significant difference in FS between the mLCMB and nmLCMB groups (*t*_(28)_ = 1.210, *p* = 0.236).

The mean scores for CDR, MMSE, and HDS-R among the 40 patients were 1.29 (SD: 0.62), 20.65 (4.96), and 18.45 (5.57), respectively. There was no significant difference in CDR, MMSE, or HDS-R score between the mCMB and nmCMB groups, or between the mLCMB and nmLCMB groups.

Serum TC in the mCMB group was significantly lower compared to that in the nmCMB group (*t*_(38)_ = 2.215, *p* = 0.033), while there was no significant difference in LDL-C/HDL-C, TG, HbA_1C_, eGFR, or BNP. The presence of hypertension in the mCMB group was significantly higher than that in the nmCMB group (*t*_(38)_ = 2.135, *p* = 0.039), while there was no significant difference between the nmLCMB and mLCMB groups (*t*_(28)_ = 1.906, *p* = 0.067). As a result of ApoE genotype evaluation, chi-squared analysis showed no significant difference in ApoE ε4 prevalence between groups.

## Discussion

EI was significantly larger in the mCMB and mLCMB groups compared to the nmCMB and nmLCMB groups, while there was no significant difference in % HGM, % BGM, and % BWM. Thus, AD patients with multiple CMB had larger lateral ventricles when compared to those without multiple CMB, however, there was no significant difference in gray and white matter volume between groups. As such, the difference in lateral ventricle enlargement in AD with and without multiple CMB not only reflects the degree of brain atrophy but also varies in the extent of CSF and ISF stasis. Calculating EI from brain MRI is the most common and simple method to assess ventricular enlargement (Brix et al., 2017). In general, lateral ventricle enlargement without DESH in dementia is conceivably due to brain atrophy. Indeed, the width of the lateral ventricle increases with age (Hashimoto et al., 2010). Although AD is a common dementia disease with characteristic brain atrophy, lateral ventricle enlargement in AD is naturally recognized as a result of brain atrophy, differing from that of iNPH. The frequent co-occurrence of AD pathology in patients with iNPH suggests a possible link between these disorders, and so enlargement of the ventricles may serve as a marker of faulty CSF clearance in AD (Ott et al., 2010). We added the VSRAD analysis, comparing the gray and white matter volume, to confirm if the difference in lateral ventricle enlargement in AD is solely a consequence of brain atrophy. This analysis suggested no significant difference in brain volume, thus the difference in lateral ventricle enlargement between AD with and without multiple CMB is presumed to reflect the not only the degree of brain atrophy but also the extent of CSF stasis.

**Figure 3:**
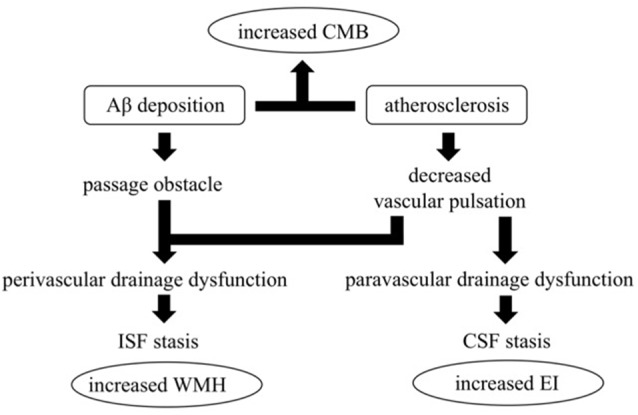
The hypothesis of impaired glymphatic circulation underlying AD: relevance of CMB, enlarged EI, and white matter hyperintensities (WMH) appearance. Impairment of the glymphatic system is an underlying pathophysiological aspect of AD. Peri- and para-vascular pathways are thought to play a crucial role, and vessel pulsations appear to be the driving force for both pathways. Vessel stiffness due to atherosclerosis reduces vascular pulsation and induces impaired peri- and para-vascular lymphatic drainage. Moreover, vascular Aβ deposition itself induces interstitial fluid (ISF) stagnation by passage obstruction. ISF stagnation results in WMH appearance and cerebral spinal fluid (CSF) stagnation results in lateral ventricle enlargement which increases EI. Both atherosclerosis and vascular Aβ deposition increase CMB.

FS was significantly higher in the mCMB group compared to the nmCMB group, indicating that WMH was more severe mCMB cases. Increased WHM is an important finding, suggesting a disturbance of ISF diffusion (Carare et al., 2013; Benjamin et al., 2014; Prins and Scheltens, 2015). It has long been assumed that WMH are due to arteriosclerotic SVD and infarction, but recently it has been proposed that WMH is the consequence of failure to eliminate ISF from white matter, an effect sometimes associated with CAA (Roher et al., 2003). The deposition of Aβ in basement membranes in CAA appears to further impede perivascular drainage (Hawkes et al., 2011). The surrounding amyloid deposits destroy vascular walls and block perivascular spaces at the level of the cerebral cortex and leptomeninges (Roher et al., 2003). Moreover, when arteries become stiffer by atherosclerosis, the amplitude of vascular pulsations will reduce. Previous studies have suggested that the motive force for perivascular drainage of ISF and solutes is derived from vascular pulsations (Carare et al., 2008; Weller et al., 2009; Iliff et al., 2013). Such a reduction in pulsations may reduce the motive force for perivascular drainage (Weller et al., 2015). Although atherosclerosis and Aβ vascular deposition are two major pathological mechanisms underlying CMB in AD, the finding that AD with multiple CMB had more severe WMH does not contradict the hypothesis that severe vascular dysfunction in AD contributes to an impairment of glymphatic drainage (see Figure 3). Obstruction and decreased pulsation results in the stasis of ISF, with simultaneous dilation of the white matter perivascular spaces (Weller et al., 2015). In contrast, there was no significant difference in FS between the mLCMB and nmLCMB groups. Although mLCMB does not reflect the atherosclerotic vascular degeneration of white matter, the glymphatic system may be less impaired compared to mCMB. The presence of WMH is also common in iNPH, a disease caused by the impairment of CSF circulation (Bradley, 2001; Johansson et al., 2016). WMH in iNPH is often reversible by shunting treatment (Tullberg et al., 2001). These results suggest that stagnation of CSF circulation also disturbs ISF circulation and that improving CSF circulation can contribute to improving ISF circulation. Thus, CSF and ISF circulation, along with peri- and paravascular lymphatic drainage, could all influence each other.

A recent glymphatic model suggests that CSF and ISF are interchanged continuously, promoting the efficient elimination of soluble proteins and metabolites from the central nervous system (Jessen et al., 2015). An influx of CSF along para-arterial (Virchow-Robin) space from the subarachnoid space is mixed with ISF and solutes, then cleared from the brain *via* para-venous spaces. CSF is driven into the para-arterial spaces by a combination of arterial pulsation and CSF pressure gradients from the subarachnoid space (Jessen et al., 2015). Efficient glymphatic clearance of waste and solutes is dependent on fluid movement across the aquaporin-4 channels located on the astrocytic endfeet surrounding the parenchymal vasculature (Bakker et al., 2016). Impairment of glymphatic system has been recently considered a common risk factor for pathological exacerbation of AD and iNPH (Golomb et al., 2000; Silverberg et al., 2003). One of the major pathological effects of impaired CSF/ISF drainage appears to be the loss of homeostasis of the extracellular environment, such that metabolites, including soluble Aβ, accumulate within brain tissue (Iliff et al., 2012; Da Mesquita et al., 2018). In recent studies, the CSF Aβ level was equally low in both AD and iNPH compared to healthy controls. The frequency of AD pathology, as shown through cortical biopsies, was found to be greater in iNPH than in the general population, suggesting that decreased Aβ clearance could be a common pathophysiological mechanism for both AD and iNPH (Cabral et al., 2011; Pyykkö et al., 2014; Pomeraniec et al., 2016; Manniche et al., 2019). Facilitating lymphatic drainage of Aβ in the aged population may prevent Aβ accumulation in the brain, maintain homeostasis, and provide a therapeutic strategy to avoid a cognitive decline in AD (Bakker et al., 2016). No significant differences in general cognitive function were detected between the mCMB and mLCMB groups, or the nmCMB and nmLCMB groups in our investigation. However, we only included simple cognitive assessments, therefore more detailed cognitive assessments will be necessary to evaluate any subtle differences in cognitive function between groups in the future.

In this study, we divided patients into non-multiple (none or one) CMB and multiple (more than two) CMB groups. The rationale behind this grouping was the modified Boston criteria for probable CAA emphasizes the presence of multiple CMB or a single CMB with cortical superficial siderosis (Linn et al., 2010). In addition, previous studies have suggested that patients with multiple CMB had more hemorrhagic complications, rather than patients with none or one CMB (Dannenberg et al., 2014).

Here, we evaluated the degree of CSF/ISF stasis in AD between patients with or without multiple CMB by comparing the degree of ventricular enlargement and WMH in brain MRI. The finding that AD with multiple CMB had greater lateral ventricle enlargement and increased WMH compared to AD without multiple CMB may reflect a difference in the impairment of the glymphatic system, especially in the peri- and paravascular pathways. Elucidating the degree of glymphatic impairment may help to predict AD progression and prognosis. EI and WMH measurement is simple and convenient, but not sufficiently detailed to evaluate CSF/ISF stagnation in AD. Recently, it has been shown that partial movement of CSF can be visualized noninvasively using an unenhanced MRI technique (Abe et al., 2014; Yamada and Kelly, 2016). In the future, further imaging techniques should be adopted to evaluate the entire CSF and ISF circulation in AD more accurately.

## Data Availability Statement

The datasets generated for this study are available on request to the corresponding author.

## Ethics Statement

The studies involving human participants were reviewed and approved by the Ethics Committee of Showa University School of Medicine. The patients/participants provided their written informed consent to participate in this study.

## Author Contributions

TK and KO contributed to the conception and design of this research. TK and MH contributed to the analysis and interpretation of the data as well as the initial drafting of the work. All authors contributed to the acquisition of the data, critically revised it for important intellectual content, and approved its final version. All authors are accountable for the contents of this research.

## Conflict of Interest

The authors declare that the research was conducted in the absence of any commercial or financial relationships that could be construed as a potential conflict of interest.
